# Improving the resistance and resilience framework for aging and dementia studies

**DOI:** 10.1186/s13195-020-00609-2

**Published:** 2020-04-14

**Authors:** Eider M. Arenaza-Urquijo, Prashanthi Vemuri

**Affiliations:** 1 Barcelonaβeta Brain Research Center (BBRC), Pasqual Maragall Foundation, Barcelona, Spain; 2 IMIM (Hospital del Mar Medical Research Institute), Barcelona, Spain; 3 Centro de Investigación Biomédica en Red de Fragilidad y Envejecimiento Saludable (CIBER FES), Madrid, Spain; 4 Department of Radiology, Mayo Clinic, Rochester, MN USA

**Keywords:** Resilience, Resistance, Alzheimer’s disease, Lifestyle, Cognitive reserve

## Abstract

**Background:**

The "resistance vs resilience" to Alzheimer’s disease (AD) framework (coping vs avoiding) has gained interest in the field in the last year. In this viewpoint, our effort is (i) to provide clarity to the usage of the framework in the context of the ATN (amyloid/tau/neurodegeneration) system as well as in lifespan and cognitive aging studies and (ii) to discuss the challenges of matching these concepts to specific biological mechanisms.

**Main body:**

In the context of the ATN system, the main goal of the resistance vs resilience framework is to make a fundamental distinction between risk factors that may help halt the development of AD pathologies (AT) (“resistance”) vs delay processes downstream to AT, i.e., neurodegeneration (N) and the clinical expression of the disease (“resilience”). The process of resilience in dementia and aging research should be envisioned as a process that is developed over the lifespan. Greater neurobiological capital to start with (initial brain reserve), maintaining brain structure and function (brain maintenance), or greater adaptability of cognitive strategies to perform a task (cognitive reserve) could all contribute to higher resilience to pathologies later in life. Simply put, resilience is not only a response to pathological processes (i.e. increased brain function to compensate for increasing AD pathology) but also reflects individual differences in brain structure and function that can be built over the lifespan (e.g., through education, lifetime cognitive, and physical activities). Further, the resistance vs resilience terminology can be extended to study other pathological processes such as cerebrovascular lesions, Lewy body disease, or TDP-43. However, some challenges do exist: (i) when studying multiple neuropathologies, the study design and framework will drive the usage of terminology; (ii) it is unavoidable that the measurements of resilience (brain structure and function) will reflect both the effect of pathologies and the impact of several risk and protective factors throughout the lifespan. Therefore, identifying resilience brain markers across lifespan, aging, and dementia studies, notably with longitudinal study designs, will be an important step towards understanding mechanisms of action.

**Conclusions:**

While the field advances towards consensus definitions of existing concepts, the resistance vs resilience terminology may provide clarity in the communication of results in aging and dementia studies as well as provide a framework for the development of both hypotheses and study designs.

## Background

The "resistance vs resilience" to Alzheimer’s disease (AD) framework (coping vs avoiding) has gained interest in the field in the last year [1, 2]. In the recent NIA-supported workshop on research definitions for reserve and resilience in cognitive aging and dementia, several points were discussed in relation to the “resistance vs  resilience” terminology that we proposed previously [1]. In this viewpoint, our effort is to bring forth clarity to these issues, specifically (i) clarifying and broadening the usage of the framework and (ii) discussing the challenges of matching these concepts to specific biological mechanisms.

## Main text

Bearing in mind our previous work, our goal has always been to propose a conceptual framework to direct research towards a better understanding of *why* and *how* some older adults remain cognitively unimpaired with high burden of neuropathologies vs absence or low burden of neuropathologies. The framework was built on existing concepts [3–9] (presented in Stern et al. [10]), highlighted their complementarity, and was structured under the umbrella terms "resistance" and "resilience" (avoiding vs coping with pathologies). “Resistance” was defined as the absence or lower-than-expected levels of AD pathology, whereas “resilience” was defined as better-than-expected cognitive performance, relative to the degree of AD pathology.

### Resilience and resistance: mechanisms and contributors

In AD research, resilience refers to the notion of “coping with pathologies” [1]. Even though we focused on resilience in AD, the term should not be dissociated from its mechanisms and lifetime contributors and can be used more broadly.

#### ATN framework

The usage of resilience can be applied in the context of the ATN scheme [11] with the rationale that brain structure and function mediate the effect of pathology on cognition. Higher brain resilience postpones the effect of AD pathology on cognition and thus delays its clinical expression . Therefore, resilience can be reflected in the variability of N (for example, less than expected cortical thinning for a given level of pathology) that is closely associated with cognitive impairment and thus explains variability in cognitive outcomes.

As pointed out above, resistance refers to lower-than-expected pathology. The usage of resistance can be applied to describe the absence of pathologies when expected, but also a delayed onset of pathology or a slower rate of accumulation.

Therefore, within the ATN scheme, resilience vs resistance can be investigated through N vs AD pathologies. Our goal within the ATN framework is to make a fundamental distinction between risk and protective factors that may help halt the development of AD pathologies (AT) (“resistance”) vs delay processes downstream of AT (i.e., neurodegeneration [N] and the clinical expression of the disease [“resilience”]), which can be translated to successful interventions. Studying resilience through brain structure and function will facilitate continuity from lifespan and aging studies. This will entail (1) extending the research on resilience in AD to systems not primarily targeted by the disease (because disease-impacted regions are often selected as measurements of N) and also (2) investigating how brain function can help compensate for increasing levels of N.

#### Lifespan and cognitive aging studies

Often, cognitive aging/dementia and lifespan studies are seen as serving distinct scientific goals. However, the process of resilience in dementia should be envisioned as a process that is developed over the lifespan. There are several mechanisms that may lead to greater resilience in later life: higher neurobiological capital to start with (initial brain reserve [7]), maintaining brain structure and function [4, 5], or showing greater adaptability of cognitive strategies to perform a task (cognitive reserve [3] or compensation see [6]). Note that these are only specific examples to illustrate the complementarity of the concepts.

While study designs are discussed in [1], we present here specific examples that can help in distinguishing between different pathways. Brain reserve (neurobiological capital) at any point in life may be a product of the initial brain reserve and maintenance of brain structure. Lifespan perspectives and longitudinal studies may help understand and disentangle these concepts and thus the mechanism(s) underlying resilience. Similarly, cognitive reserve could be studied as an initial difference in cognitive processes but may be better recognized when measurements of pathology are available. Further, we believe that investigating the maintenance of brain structure and function in the presence of pathologies is an important area of research. For example, exercise may help maintain brain structure and cognition in the face of amyloid [12]. This notion is different from “general” brain maintenance [4] and refers to the maintenance of specific brain processes (see also [5, 6]).

It is important to note that resilience is not only a response to a pathological process as defined by cognitive reserve and compensation (i.e., increased brain function to perform a task and compensation for increasing AD pathology) but also refers to individual differences in brain structure and function that can be built over the lifespan through education, lifetime cognitive, and physical activities among others.

#### Broadening the usage to other pathologies

While our original intent was to provide terminology for preclinical AD studies, the terminology can be easily extended to other pathological processes. The conceptualization of resistance vs resilience in the context of a lesion as done by Montine and colleagues is useful in this regard [2]: they define resistance as an observed lower level of brain injury associated with dementia and resilience as an observed level of cognitive functioning higher than expected in the face of brain injury.

We can adopt a similar approach and explain this extension of usage in the context of our original work [1] using three sets of variables: (1) a measure of pathology, (2) a measure of a protective factor/mechanism, and (3) a measure of cognition. Pathology (“brain injury” in Montine et al. paper) can be broadened to include cerebrovascular lesions, Lewy body disease, and TDP-43. For example, higher white matter integrity (resilience mechanism) may provide resilience in the presence of cerebrovascular lesions (brain injury or pathology).

### Tackling the issue of matching concepts to mechanisms

#### Mapping resilience vs resistance to a specific process

The resistance vs resilience terminology can be agnostic to specific scientific hypotheses. For example, in the context of the amyloid cascade, resistance to tau could be operationalized in the face of amyloid positivity. However, it is also possible to study factors that explain lower-than-expected tau in the context of aging. The term “resistance to tau” means lower tau than expected and can be utilized in both these examples.

In the specific context of AD research and the amyloid cascade hypothesis, the differentiation between resilience and resistance to amyloid and tau has been debated. It is widely understood that the presence of amyloid drives increased tau deposition which in turn significantly increases neurodegeneration and ultimately cognitive impairment. In an example study where higher gray matter volume for a given level of amyloid is observed, one could use “resilience to amyloid” if preservation of brain structure or lower rate of atrophy associated with a protective factor was observed, or “resistance to tau” if lower tau was seen at the same level of amyloid (explaining the greater gray matter volume). Most importantly, when measurements of multiple pathologies are not available, the scientific framework of the study will drive the usage of the terminology. The notion of “apparent resilience” proposed by Montine et al. is useful to bear in mind the limitation of the usage of resilience when co-pathologies are not measured. For example, in the context of the amyloid hypothesis, in a study design where modifiers for tau deposition are investigated accounting for amyloid deposition, then resistance to tau would be the most appropriate terminology to use. However, if the study design remains agnostic to a temporal ordering of mechanisms underlying AD pathogenesis, resistance to tau and amyloid may be studied separately.

Nevertheless, it is important to be clear that resilience is not a pathological response to another pathology (i.e., amyloid driving tau) but a brain mechanism that helps either avoid or cope with the effects of pathology on cognition. The study by Perez-Nievas and colleagues showing preservation of neuron number and synaptic markers in non-demented older adults with high amount of plaques and tangles at autopsy illustrates the correct usage of resilience [13].

Finally, we acknowledge the difficulty of studying resilience in imaging and biomarker studies because pathologies directly affect brain structure and function and thus measurements of resilience. This would be especially true in cognitively impaired participants where neurodegeneration will be more extensive, and measurements of resilience may reflect both pathological processes and the impact of several risk and protective factors throughout the lifespan. Longitudinal studies with measurements of brain structure and function and multiple pathologies will be fundamental to address this issue. If the framework was to be extended to cognitively impaired participants, the inclusion of multiple cognitive measurements will be especially important for studying these concepts. Identifying brain signatures or markers of resilience across studies will be an important step . For example, several studies have provided converging evidence of the involvement of lateral frontal and medial prefrontal regions supporting resilience at older ages (see for example [14, 15]).

## Conclusions

Consensus is needed for the operationalization of these concepts in a unified way, as well as to match them to specific biological mechanisms. While the field advances in this direction, the resistance vs resilience terminology can help with more precise communication of the results and the framework can aid in the development of specific hypotheses and study designs.

## Data Availability

Not applicable.
